# Barriers to Adoption of Mineralocorticoid Receptor Antagonists in Patients With Heart Failure: A Mixed‐Methods Study

**DOI:** 10.1161/JAHA.115.002493

**Published:** 2016-03-31

**Authors:** Sandesh Dev, Trisha K. Hoffman, Dio Kavalieratos, Paul Heidenreich, Wen‐Chih Wu, Dawn C. Schwenke, Sarah J. Tracy

**Affiliations:** ^1^ Cardiology SectionPhoenix VA Health Care SystemPhoenixAZ; ^2^ Research SectionPhoenix VA Health Care SystemPhoenixAZ; ^3^ Hugh Downs School of Human CommunicationArizona State UniversityTempeAZ; ^4^ Section of Palliative Care and Medical EthicsUniversity of PittsburghPittsburghPA; ^5^ Cardiology SectionPalo Alto VA Medical CenterPalo AltoCA; ^6^ Cardiology SectionProvidence VA Medical CenterProvidenceRI

**Keywords:** qualitative research, quality of care, spironolactone, Heart Failure, Cardiomyopathy, Quality and Outcomes, Pharmacology

## Abstract

**Background:**

Mineralocorticoid receptor antagonists (MRAs) are the most underutilized pharmacotherapy for heart failure. Minimal data are available on the barriers to MRA adoption from the perspective of prescribing clinicians.

**Methods and Results:**

A mixed‐methods study consisting of a survey (n=50), focus groups (n=39), interviews (n=6) with clinicians at a single US Department of Veterans Affairs medical center served to ascertain barriers to optimal use of MRAs. Participants were drawn from 6 groups: cardiology providers, cardiology fellows, hospitalists, clinical pharmacists, internal medicine residents, and primary care providers. Qualitative data were iteratively coded with qualitative data analysis software. The survey response rate was 17.3%. Overall, 51% of survey respondents were unfamiliar with eplerenone, and 6% were unfamiliar with spironolactone. In addition, 30% of respondents reported that they would order a laboratory test >2 weeks after a new MRA prescription, although that is beyond the guideline recommendation. Most providers correctly identified New York Heart Association class 3 and 4 patients as MRA eligible, but only 42% identified class 2 patients as MRA eligible. Through analysis of focus groups, we identified 8 barriers to MRA use in 3 categories: patient‐based barriers (concerns about polypharmacy and comorbidities, adverse effects, perceived patient nonadherence), provider‐based barriers (unclear roles and responsibilities, coordination and transitions of care, lack of experience or familiarity with MRAs), and system‐based barriers (system overload and provider time constraints, lack of systematic follow‐up procedures).

**Conclusions:**

Eight primary barriers to MRA adoption at the provider, patient, and health system levels were identified from the prescriber perspective. These barriers can inform the creation of multilevel interventions that will be required to close the gap in MRA adoption.

## Introduction

The management of heart failure (HF) poses a tremendous burden on patients, caregivers, and the health system. HF accounts for nearly 1 million annual hospitalizations in the United States^1^ and >3 million physician office visits.^2^ With expected improvements in survival and the aging of the population, experts project that by 2030, 1 in every 33 US citizens will have a diagnosis of HF.^3^ Consequently, it is imperative that cost‐effective and high‐value therapies are optimally implemented.

Mineralocorticoid receptor antagonists (MRAs), most commonly spironolactone, are a class of drugs shown in randomized clinical trials to markedly reduce mortality, hospitalization, and sudden death in patients with HF and reduced ejection fraction.^4^, ^5^, ^6^ Prior studies, however, found that adoption of MRAs^7^, ^8^, ^9^ was poor in outpatients with HF and in HF patients after hospitalization. In a national sample of >12 000 patients hospitalized for HF who were candidates for an MRA, only one‐third received a prescription at discharge. Similarly, safety monitoring for hyperkalemia, a common adverse event of MRAs, is inadequate. In a sample of 122 US Department of Veterans Affairs (VA) hospitals and >175 000 patients with a diagnosis of HF, the hospital‐level average for patients receiving potassium monitoring within 14 days after initiation of an MRA was 37%.^10^ Despite recognition of this gap, there is a dearth of empirical evidence regarding the reasons for low utilization and poor safety monitoring of MRAs. We used a mixed‐methods design to describe barriers to MRA use from the perspective of front‐line prescribing clinicians.

## Methods

This study consisted of 3 sequential phases: (1) a quantitative survey, (2) focus group interviews, and (3) “member check” interviews—a common qualitative practice for increasing the credibility and rigor of findings. The Phoenix VA Health Care System institutional review board approved the study, and participants provided informed consent. A multidisciplinary team of investigators including VA insiders and outsiders and quantitative and qualitative experts developed the study design. The existing literature on barriers to adoption of practice guidelines informed the survey and focus group topics.^11^

### Survey

We recruited a convenience sample of providers with the potential for association with MRA prescribing by inviting all providers, including resident physicians, from inpatient internal medicine, cardiology, and primary care at the Phoenix VA Medical Center (Table 1). Participants were recruited by telephone, by email, and in person and completed the survey (Table 2) using an Internet‐based data collection system (Qualtrics). The rationale behind the survey was to provide information about the practices and the knowledge of focus group participants so that we could more effectively design the focus group. Participants were asked to complete the survey if they were interested in participating in a subsequent focus group.

**Table 1:** Characteristics of Study Participants

Total Number of Providers Within Scope of Study (n=53)	Results, n (%)
Survey participation only	8 (15)
Survey and focus group	39 (74)
Survey and focus group and interview	3 (6)
Interview only	3 (6)
Characteristics of survey participants (n=50)	
Department	
Primary care	11 (22)
Pharmacy	13 (26)
Internal medicine	13 (26)
Cardiology	12 (24)
Other	1 (2)
Professional title	
Staff physician	15 (30)
Midlevel (NP or PA)	7 (14)
Resident physician	14 (28)
Pharmacist	14 (28)
VA primary work location	
Main hospital	43 (86)
Community clinic—metro area	4 (8)
Community clinic—rural	3 (6)
Total years in practice (including residency), median (IQR)^a^	9 (3–15)
Years in practice at Phoenix VA (including residency),^a^ median (IQR)	6 (1–10)

Results are shown as number (percentage) or median (IQR), as noted. IQR indicates interquartile range; NP, nurse practitioner; PA, physician assistant; VA, US Department of Veterans Affairs.

a Indicate n=49 for both questions.

**Table 2:** Survey Questions and Provider Responses

Question	Results, N=50 n (%)
“It is the responsibility of the Cardiology Division, and not Internal Medicine or Primary Care to initiate aldosterone antagonists for HF patients”	
Strongly disagree	10 (20)
Disagree	27 (54)
Agree	8 (16)
Strongly agree	5 (10)
Please rate your familiarity with aldosterone antagonists	
Spironolactone	
Completely unfamiliar	0 (0)
Not very familiar	3 (6)
Familiar	22 (44)
Very familiar	24 (48)
No response	1 (2)
Eplerenone	
Completely unfamiliar	6 (12)
Not very familiar	19 (38)
Familiar	20 (40)
Very familiar	4 (8)
No response	1 (2)
“Based on your knowledge of aldosterone antagonists (spironolactone and eplerenone), which of the following are known side effects or contraindications to aldosterone antagonists therapy?”	
Uncontrolled hypertension	0 (0)
Bradycardia	1 (2)
Hyperkalemia	48 (96)
Cough	0 (0)
No response	1 (2)
“Based on your knowledge of aldosterone antagonists (spironolactone and eplerenone), what is the main difference in side effect profile between the two drugs?”	
Uncontrolled hypertension	0 (0)
Breast enlargement	46 (92)
Bradycardia	1 (2)
Allergy to drug	1 (2)
Hyperkalemia	1 (2)
No response	1 (2)
“Based on your experience, are aldosterone antagonists easy or difficult to prescribe?”	
Very difficult	0 (0)
Difficult	4 (8)
Easy	34 (68)
Very easy	11 (22)
No response	1 (2)
“Based on your experience, are aldosterone antagonists easy or difficult to monitor with lab testing?”	
Very difficult	0 (0)
Difficult	7 (14)
Easy	38 (76)
Very easy	5 (10)
“After you write a new prescription for aldosterone antagonist, based on your experience, when would you order a follow‐up test for monitoring?”	
≤2 weeks	35 (70)
≤1 month	13 (26)
≤2 months	2 (4)
>2 months	0 (0)
“Which NYHA HF classes are eligible for an aldosterone antagonist?” (choose all that apply)	
NYHA class 1	6 (12)
NYHA class 2	21 (42)
NYHA class 3	43 (86)
NYHA class 4	39 (78)
“Indicate the maximum left ventricular ejection fraction in which you would start an aldosterone antagonist (0–70),” median (25th–75th)^a^	40 (35–40)
“Indicate the maximum serum creatinine in men in which you would start an aldosterone antagonist (1 decimal place),” median (25th–75th)^a^	2.0 (1.5–2.5)
“Indicate the maximum serum creatinine in women in which you would start an aldosterone antagonist (1 decimal place),” median (25th–75th)^a^	1.8 (1.5–2.0)
“Indicate the minimum glomerular filtration rate (eGFR) (mL/min per m^2^) in which you would start an aldosterone antagonist (men and women) (0–125 mL/min per m^2^),” median (25th–75th)^b^	30 (30–40)
“Indicate the maximum serum potassium in which you would start an aldosterone antagonist (men and women) (0–10) (max 1 decimal place),” median (25th–75th)^a^	4.9 (4.5–5.0)
“Patient should be on beta blocker therapy, if eligible”	
Agree	48 (96)
Disagree	1 (2)
No response	1 (2)
“Patient should be on ACE inhibitor or angiotensin receptor blocker, if eligible”	
Agree	47 (94)
Disagree	1 (2)
No response	2 (4)
“From the choices below, drag and drop in the box ‘potential barrier’ between 0 and 3 barriers that you believe may limit the use of aldosterone antagonists in HF patients”	
Just being aware of the drugs	8 (16)
Your own familiarity with the drugs	16 (32)
Your own agreement with specific drug guidelines	1 (2)
Your agreement with guidelines in general	0 (0)
Being able to perform the guideline recommendation	4 (8)
Believing the drug will improve the desired outcome	3 (6)
Feeling motivated and feeling as if it is routine to prescribe these drugs	4 (8)
Patient preferences	8 (16)
Environment (enough time, resources, organizational opportunities, reimbursement, liability)	7 (14)
Potential for side effects	28 (56)
Concern regarding starting ACE and beta‐blocker first	18 (36)
Ease of monitoring	6 (12)
The number of drugs for HF and other conditions (polypharmacy)	27 (54)

Results are shown as number (percentage) or median (25th–75th quartiles), as noted. ACE indicates angiotensin‐converting enzyme; HF, heart failure; max, maximum; NYHA, New York Heart Association.

a Indicate n=46 for these three questions.

b Indicate n=47 for both questions.

The survey gauged participants' familiarity with MRAs, especially spironolactone; knowledge of appropriate prescription and monitoring practices; perception of potential barriers that could limit the use of MRAs in HF patients; and perception of effective interventions that would increase MRA use, appropriate monitoring, and appropriate prescription.

Survey findings were described as proportions for categorical variables and as median (interquartile range) for continuous variables. To gain insight into the knowledge and attitudes of subgroups of participants, we conducted an exploratory analysis (chi‐square test) in which responses were analyzed by clinical department (primary care, inpatient internal medicine, cardiology, and pharmacy), years of experience (tertile), and professional title (physician, nurse practitioner, or pharmacist). Given the small sample size, we decided a priori that any findings with *P*<0.10 could indicate potential significance. Questions with responses on a 4‐point Likert scale were dichotomized to 2 responses to simplify analysis. We then tested for an association between knowledge of MRA side effects and familiarity with spironolactone and eplerenone, respectively. We also tested for an association between knowledge of MRA side effects with knowledge of MRA indications; appropriate laboratory‐monitoring intervals; and thresholds for renal function, potassium, and left ventricular ejection fraction (Fisher exact test, significance level of 0.05).

### Focus Groups

Given the paucity of knowledge regarding MRA utilization, focus groups were used to further explore the phenomenon of MRA prescription within the VA. In contrast to individual interviews, focus groups allow dynamic interaction and collective learning among similar participants.^12^ Of the survey participants, 42 elected to participate in focus groups (ie, 84% recruitment rate) (Table 1). We convened 6 focus groups, stratified by stakeholder category so as to develop a comfortable group atmosphere and encourage discussion of shared experiences: (1) cardiology nurse practitioners and cardiologists, (2) cardiology fellows in training, (3) hospitalists, (4) clinical pharmacists, (5) internal medicine residents, and (6) primary care physicians and nurse practitioners. We developed a semistructured focus group guide based on the extant literature regarding barriers to MRA prescription and a preliminary analysis of the aforementioned survey data. Focus groups each lasted 2 hours and were facilitated by a qualitative researcher (T.K.H.) and a cardiologist (S.D.), each trained by an expert qualitative methodologist (S.J.T.). Focus groups included a mix of open‐ended question‐and‐answer discussion, free writing, brainstorming exercises, and polling—exercises that allowed for flexibility but allowed facilitators to ask specific questions about the most significant barriers to MRA use, safety‐monitoring strategies, and prescription strategies.

### Member Check Interviews

Focus groups were followed by interviews of 6 participants who possessed experience regarding MRA prescription and monitoring (Table 1); 2 interviewees had participated in focus groups and 4 had not. These participants included a pharmacy director, a VA HF outcomes researcher/cardiologist, a home care specialist, a hospitalist/residency program director, a cardiology nurse practitioner, and a pharmacy informaticist. Engaging in these type of “member check” interviews^13^ allowed the research team to corroborate findings and assess their potential relevance for medical professionals beyond those who participated in the study.^13^ This technique and others used for enhancing the rigor of our qualitative methods are listed in Table 3.

**Table 3:** Practices That Ensured Meeting Criteria for Qualitative Quality^13^, ^14^, ^15^, ^16^

Criteria	Practices and Methods
Worthy Topic *Is the topic relevant, timely, significant, and interesting?*	The study addressed a major problem of heart failure and the underutilization of an effective drug. Authors continually revisited the literature so as to build on past research and provide significant conclusions.
Rich Rig or*Does the study use sufficient, abundant, appropriate and complex theory, data, sampling techniques, and analysis processes?*	Disciplinary experts reviewed and helped develop study instruments. Data were abundant—from 53 participants, using 3 different sources of data (survey, focus group, interview), over the course of 13 months, resulting in 276 type‐written single‐spaced pages of transcripts. Authors engaged in purposeful and targeted sampling (maximum variation; grouping of similar participants in focus groups; expert informants for interviews). Findings emerged via grounded and incremental development of methodological instruments. Surveys were developed from existing literature; focus group guides were developed from survey data and literature; interview questions were developed from the survey and focus group data. This approach created consistency and built on prior knowledge. Analysis was iterative in nature, moving between emergent open coding, analytic memo writing, the development of code structures, and used Nvivo 10 qualitative data analysis software.
Sincerity and Ethics *Is the study characterized by transparency and ethics?*	The research team included both health care insiders and outsiders so as to mitigate the potential vested interests of promoting a certain drug Bracketing^17^ allowed for recognition of investigators' preconceptions and assumptions The article shares information about methodological challenges and study limitations The research passed human subject approval; any identifiable data has been collapsed in the publication Participants were offered reports of the data as a method of “exiting ethics”
Credibility and Plausibility *Are the findings trustworthy and dependable?*	The study triangulated data sources (survey, focus group, and interview). Focus groups and interviews were professionally transcribed and checked for accuracy by the research team. The study used a plurality of voices from 6 different stakeholder populations. Member check interviews with expert informants provided increased credibility. Authors maintained an audit trail throughout the analytic process, detailing decision rules and analytic directions. The research team held frequent debriefings to discuss findings and concerns.
Resonance and Transferability *Are the findings applicable to other contexts or situations?*	A detailed literature review provided the context within which our work falls. In the conclusion of the article, the research team provided direction about the extent to which the findings could be adopted in other contexts.

Interviews lasted ≈20 minutes. Interviewees were given a summary of the predominant MRA barriers and asked (1) whether the summary reflected the primary barriers to MRA prescription and monitoring; (2) to identify which were the top 3 barriers, in their opinion, and why; (3) whether there were any barriers on the list that seemed problematic or inaccurate and why; and (4) whether there were any barriers they felt were missing and, if so, to explain.

### Qualitative Analysis

Focus groups and interviews were audiotaped and transcribed, and the first author spot‐checked the transcripts against the recordings for accuracy. The focus groups resulted in 195 pages of single‐spaced transcripts, and the interviews generated 81 pages of single‐spaced transcripts. To identify overall barriers experienced across the variety of providers, the data from all 6 focus groups were read and coded together with the guiding question, “What are the barriers to spironolactone [MRA] use?” We noted when the data contrasted between provider subgroups (eg, cardiologists versus primary care providers [PCPs]). Using an iterative approach,^13^ similar chunks of information were organized into larger conceptual bins, and codes were given definitions. We developed analytic memos describing coding decisions and implications. Throughout the analytic process, the authors consulted previous literature and examined how the emergent data differed from and/or reiterated barriers identified in previous studies.

We developed a code book that detailed the barriers to spironolactone use. As an organizing scheme, the barriers were grouped together as patient barriers (eg, adverse drug effects), system barriers (eg, lack of systematic follow‐up procedures), and provider barriers (eg, coordination and transition of care). We coded interview data using Nvivo qualitative data management software (QSR International). Intercoder reliability checks ensured consistent coding using a process of 2 authors independently coding 25% of the data, comparing results, and discussing discrepancies. The coding scheme was refined until a consistent, reliable, and inclusive set of coding guidelines was reached, at which time the remaining data were coded by the second author. We met frequently throughout the analytic process to discuss coding. We calculated the number of words associated with each code to gauge the amount of talk time associated with each barrier (Table 4).

**Table 4:** Overview of Barriers Discussed in Focus Groups

Perceived Barrier	Description	Sample Quote	Focus Group Words Related to This Barrier (%)
Patient‐based			40
Patient polypharmacy and comorbidities	Providers are hesitant to add MRA when patients are on multiple medications and having multiple health issues	“Many of our patients [with] HF … they're already on 5, 10, 15, 20 medications … [and] need to have a fairly significant reason for starting a new medication.” (PCP)	17
Adverse effects of drug therapy	Providers are concerned, especially about patients who do not complete lab work, regarding the potential side effects associated with MRA, namely, hyperkalemia	“When the creatinine starts creeping up, [I am] concerned that the patient may not follow up for labs or for an office appointment … they're on a medication that raises their potassium [such] that they could prematurely die.” (Cardiology fellow)	14
Perceived patient nonadherence	Providers are concerned about patients' abilities and willingness to complete the necessary lab work follow‐up appointments when on an MRA or to take their medication consistently	I think it is specific to the patient … [If] they go to their appointments, they get the labs done as ordered versus someone who most of their encounters are either … hospitalizations or emergency department, then I'm definitely a lot less likely to start [an MRA] … We have a high population of patients who are homeless … they may want to follow up, but they're just not able to.” (Hospitalist)	9
Provider‐based			35
Unclear provider roles and responsibilities	Some providers noted that providers may defer treatment of HF to cardiology specialists but that all providers should be responsible for treating and overseeing HF and prescribing an MRA if it is considered an effective treatment	“I think … too many cooks in the kitchen and too many people are doing too many different things. … Cardiology should then maybe run the show in regards to the HF. [At the same time], if you've someone in your clinic and it's time to start the aldosterone antagonist, then I wouldn't see any reason why [any provider] wouldn't.” (Hospitalist)	16
Coordination and transitions of care	Monitoring of HF patients across departments can be difficult to maintain. Communication among providers (ie, pharmacists, cardiology, PCPs, hospitalists) can be unclear, making it difficult to prescribe MRAs or monitor patients.	“When we're titrating up a drug … that may not be communicated in the note form … so there may be reluctance to start a new medication or titrate up the dose because one hand may not know what the other hand is doing.” (Two hospitalists)	10
Lack of familiarity or experience with MRA use	Noncardiology providers described having less experience, familiarity, or comfort using MRAs. It is not a drug they commonly use, and they might experience a lack of knowledge about prescribing, monitoring, or using MRAs.	“I'm still learning to be comfortable with spironolactone, and in primary care, I don't think I would have had that kind of knowledge. … I certainly could get into that knowledge, but … I think in primary care, a lot of times we don't think in terms of that the [HF] guidelines are meant for us, honestly, but are meant for cardiology.” (Cardiology NP)“These aren't drugs that you're going to receive education about … on a regular basis, especially when it's applied just to HF, so they're not going to be at the top of somebody's list. … They're thinking valsartan or one of the new ARBs or something that is constantly … being advertised and promoted to them.” (Clinical pharmacist)	9
System‐based			25
System overload and provider time constraints	Both patients and providers may experience difficulties prescribing and taking or monitoring MRAs because of difficulties encountered in the VA system. Some providers, namely, PCPs, also noted issues with monitoring when they have high patient caseloads.	“I think they're all kind of interrelated … in the sense that primary care doesn't have the time to do it [monitor], the hospitalists may initiate it in the hospital, but then it's up the primary care to pick it up and keep it up, and that means the patient has to get in to see the PCP … within 2 weeks and that's not always happening.” (Cardiology NP)“You can order labs, but no one is really going to see or follow up with it in a timely fashion; that may prompt you to be a little more hesitant prescribing medications that have adverse effects rather in another setting.” (Cardiology fellow)	13
Lack of systematic follow‐up procedures	Data suggest lack of a clear, systematic plan for consistent follow‐up with patients on MRAs.	“They [patients] come back to us 6 months later for things that could've been avoided.” (Hospitalist)	12

ARB indicates angiotensin receptor blocker; HF, heart failure; MRA, mineralocorticoid receptor antagonist; NP, nurse practitioner; PCP, primary care provider; VA, US Department of Veterans Affairs.

## Results

### Survey

The survey response rate was 17% (50 of 294 participants). From the range of possible choices, the most commonly cited barriers to MRA use were (1) potential for side effects (56%), (2) polypharmacy (54%), (3) concern about starting an angiotensin‐converting enzyme (ACE) inhibitor (or angiotensin receptor blocker) and beta‐blocker first (36%), and (4) lack of familiarity with MRAs (32%) (Table 2). Overall, 26% of all respondents felt that cardiology specialists should initiate MRAs for HF patients. Inpatient hospitalists and residents (85%) and pharmacists (92%) did not think that cardiology providers were solely responsible for MRA initiation compared with cardiology providers (50%) and PCPs (14%; *P*=0.0815, overall comparison). Moreover, 51% of overall respondents were unfamiliar with eplerenone, and 6% were unfamiliar with spironolactone (*P*=0.23). Cardiology (75%) and pharmacy (69%) providers were very familiar or familiar with eplerenone, followed by internal medicine practitioners (41.7%); PCPs (9.1%) were not familiar with eplerenone (*P*=0.0048, overall comparison). Respondents were well aware of MRA‐related adverse effects, namely, hyperkalemia (96%) and gynecomastia (92%). Most respondents reported that MRAs were easy or very easy to prescribe (90%) and to monitor with laboratory testing (86%). Nevertheless, 30% of respondents reported that they would order a laboratory test >2 weeks after a new MRA prescription, whereas the guideline recommendations are within 1 week. Although most correctly identified New York Heart Association class 3 and 4 patients as MRA eligible, less than half (42%) identified class 2 patients as eligible. Respondents accurately identified eligibility for MRA based on left ventricular ejection fraction and serum potassium; however, when asked about the serum creatinine level at which they would initiate an MRA, their responses for men (median response 2.0 mg/dL) and for women (median response 1.8 mg/dL) were lower than the guideline‐recommended maximum serum creatinine level (<2.5 mg/dL in men, <2.0 mg/dL in women). When asked a similar question regarding the minimum estimated glomerular filtration rate at which they would start an MRA, participants' median response (30 mL/min per m^2^) was identical to the guideline recommendation.

There was no significant relationship between “knowledge of differences in side effects (gynecomastia)” between spironolactone and eplerenone and “familiarity with these medications.” Similarly, there was no significant relationship between “knowledge of MRA side effects (hyperkalemia)” and “familiarity with eplerenone” (Fisher exact test, all 2‐sided *P*=1.00); however, there was a suggestion of a posi‐tive relationship between “knowledge of MRA side effects” and “familiarity with spironolactone” (Fisher exact test, 2‐sided *P*=0.063). There was no significant relationship between “knowledge of side effects (known side effects of MRAs or differences in side effects)” and “knowledge of MRA indications (baseline beta‐blocker and ACE inhibitor use),” “lab monitoring intervals,” or “thresholds for creatinine, estimated glomerular filtration rate, serum potassium, or left ventricular ejection fraction” (Fisher exact test, most 2‐sided *P*>0.5).

### Focus Group and Member Checking Interviews

We identified 8 barriers to MRA use from 3 general sources: patient‐based, provider‐based, and system‐based barriers (Table 4). Our data indicated that providers' choice not to prescribe MRAs may be related to several barriers rather than to a single cause. Providers noted that these overlapping hurdles create risks for successful MRA use that outweigh potential benefits. Through the member checking exercise, we found support for the barriers previously identified in the focus groups.

The following findings, organized by sources of barriers, described the central themes of each barrier in detail (Figure). Given the focus group design of our study, which capitalized on the collective thinking of participants, the themes presented generally reflect the experiences of multiple participants within and across focus groups. In Table 4, we provided sample quotations to illustrate the key barriers identified by the providers.

**Figure 1:**
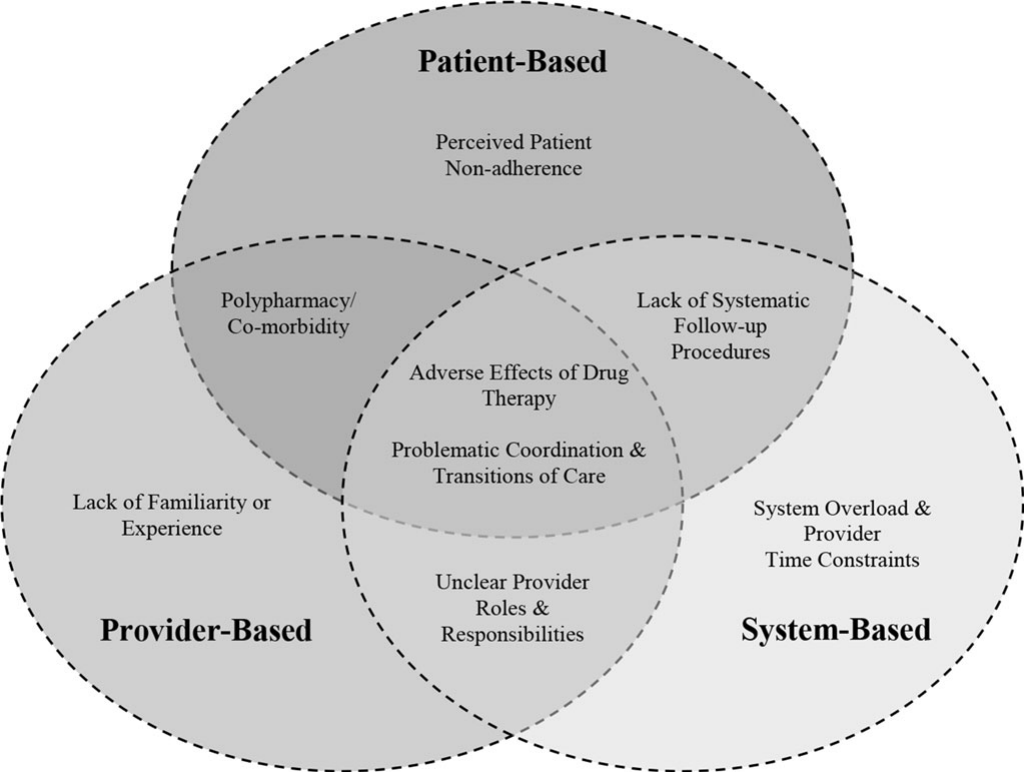
Model of barriers to MRA use. Based on focus group findings, we identified 8 barriers to MRA use from 3 general sources: patient‐based, provider‐based, and system‐based barriers. Our data indicate that providers' choice to not prescribe MRAs may be related to several barriers rather than to a single cause. MRA indicates mineralocorticoid receptor antagonist.

### Patient‐Based Barriers

#### Patient polypharmacy and comorbidities

Patient polypharmacy and comorbidities were often mentioned during discussions of adverse effects of drug therapy. Providers mentioned the number of patients' other medications as a barrier to MRA use. Many patients take multiple medications for several chronic health conditions. Providers described their hesitance to add an MRA to the mix because they believed patients already had difficulty adhering to drug therapies or they wanted to respect the preferences of patients who indicated that they did not want additional medications. Furthermore, providers cited prioritizing more pressing health issues because of a high volume of patient cases.

#### Adverse effects of drug therapy

Given the potentially fatal effect of MRA‐induced hyperkalemia, providers discussed a commitment to “do no harm” to their patients. Their fear of potential drug interactions as a result of patient polypharmacy and comorbidity overlapped with concerns about the overloaded health system that might prevent regular follow‐up, the heavy PCP caseloads, and the additional effort required to monitor MRA therapy. Patients were described as “fragile” because of the numbers of health and personal issues, and participants cited concerns about prescription due to MRA‐related changes in kidney function (ie, creatinine levels).

#### Perceived patient nonadherence

Providers referred to patient habits, lifestyles, and lack of resources as perceived barriers to adherence. Lack of patient self‐efficacy, health education, transportation to laboratory appointments, and support networks, coupled with unhealthy lifestyles and comorbidities, were cited as factors that prevent appropriate patient follow‐up. Collectively, providers expressed serious doubt about their patients' abilities to maintain the monitoring requirements necessary for safe MRA use. Many providers explained that their decision to prescribe an MRA would depend on their assessment of whether a patient could successfully follow the treatment plan.

### Provider‐Based Barriers

#### Unclear provider roles and responsibilities

Across focus groups, participants agreed that providers of all types were ultimately responsible for treating and managing HF; at the same time, noncardiologists viewed HF as a “specialty” condition that may require specialized care. Providers were concerned about overstepping professional practice boundaries and negotiating ill‐defined provider roles and expectations. In addition, there seemed to be a general consensus across groups that cardiologists should be involved in monitoring patients who are prescribed an MRA, even if another provider prescribed the drug.

#### Coordination and transitions of care

Providers explained that the difficulties of coordinating care across disciplines is a barrier to MRA use. This concern was expressed as “too many cooks in the kitchen” and highlights the challenge of managing patient care in a large‐scale health care system and teaching hospital. Challenges with provider coordination are exacerbated during transitions of care, such as transition from hospital to home or as trainees rotate to other locations. When these transitions occur, the guidelines for appropriate handoffs between providers may not always be clearly communicated or simply may not exist. Given the uncertainty of follow‐up care, providers were hesitant to start patients on a new drug that may not receive the proper follow‐up monitoring needed for safe use.

#### Lack of familiarity or experience with MRA use

Cardiology providers expressed comfort working with MRAs and demonstrated a strong working knowledge of the appropriate guidelines for prescription and monitoring; however, noncardiology providers, especially PCPs, felt they did not have the appropriate level of knowledge or expertise to use this perceived specialty drug, despite the fact that cardiologists see only half of all HF patients in the VA healthcare system (Paul Heidenreich, MD, personal communication, 2015).

Participants correlated inadequate knowledge of MRA prescription and monitoring procedures with PCPs' tendency to defer HF treatment to cardiology providers. Furthermore, they noted that noncardiology providers may not have regular contact with HF patients, and this, in turn, could decrease the likelihood of a strong working knowledge of the recommended guidelines for MRA use. Beyond these matters, providers cited a lack of familiarity with MRAs because they are not promoted like other drugs.

### System‐Based Barriers

Providers from all stakeholder groups identified the design and functioning of the hospital system as a barrier to MRA prescription and monitoring. These barriers included an overloaded hospital system, provider time constraints, and a lack of systematic follow‐up procedures for monitoring HF patients on MRAs. Beyond the lack of these clear procedures was also an indication that both overloaded provider caseloads and patient access to their PCPs inhibited use of these drugs for eligible patients and the ability to properly monitor patient compliance with follow‐up and laboratory testing.

#### System overload and provider time constraints

Providers, especially PCPs, frequently cited time constraints as a barrier to MRA use. Many providers noted the large clinical panel sizes and the comorbidities they are trying to manage in their patients. Consequently, HF tends to be a low priority for noncardiology providers because of other more pressing health issues, such as diabetes, titrating other medications, or managing acute and exacerbated conditions. Participants also reported issues with MRA laboratory‐monitoring procedures as a result of an overloaded hospital system. Because providers are aware that patients often face difficulties seeing their physicians in a timely manner, providers often choose to forgo prescribing an MRA.

#### Lack of systematic follow‐up procedures

Participants also described the lack of tools and protocols in place within the system for monitoring patients on MRAs. With patients who see multiple providers, providers are uncertain of who will be responsible for maintaining safety monitoring (ie, unclear provider roles and problematic communication). This is especially true for hospitalists, who are concerned with patient monitoring once patients transition to outpatient status. In such cases, providers were uncertain about protocols for how monitoring would be continued if patients were started on an MRA while admitted to the hospital. Participants also described a lack of reminder tools or alerts to notify them when labs had not been completed by patients. Many providers noted the high rates of missed follow‐up appointments for lab work. Although some clinics attempt to remind patients of their upcoming appointments, many patients still do not show up, and providers receive no notification when a patient's lab work was not completed. The result of the lack of follow‐up is often patient readmission to the hospital. As a hospitalist explained, “They [patients] come back to us 6 months later for things that could've been avoided.”

## Discussion

The underuse of MRAs has been identified as a critical gap in adoption of evidence‐based HF care. Studies have reported that among Medicare beneficiaries hospitalized with HF, only 1 in 4 MRA‐eligible patients receives an MRA,^18^ and among MRA‐ineligible patients, as many as 1 in 6 receives an MRA.^8^ Furthermore, safety monitoring to prevent hyperkalemia and renal failure continues to be inadequate: Only 1 in 3 patients receives serum potassium or creatinine monitoring within 14 days of prescription despite recommended monitoring within 1 week of MRA initiation.^19^ Despite the existence of these gaps in MRA utilization, the underlying reasons for them have been poorly understood. To our knowledge, this study is the first to systematically investigate barriers to MRA use by establishing a framework of patient‐, provider‐, and system‐based barriers to MRA adoption.

In 2009, a large registry‐based study documented low rates of MRA prescription in a national cohort of patients hospitalized with HF.^7^ The authors suggested that physician knowledge, familiarity, and agreement with guidelines were the primary reasons for failure to prescribe MRAs. In support of that hypothesis, we identified specific gaps in provider knowledge regarding guidelines for appropriate MRA use, specifically, the appropriate time interval for potassium monitoring and the newer recommendation of MRA use in patients with mild HF symptoms (ie, New York Heart Association class 2). In addition, during focus groups, PCPs cited a lack of experience using MRAs; this was supported by our survey, which found that only 9% of PCPs were familiar with eplerenone, a nonformulary drug in use at the Veterans Health Administration. It appears that MRA use is not part of the cultural norm for noncardiology providers in this system, creating a cyclical problem. Because PCPs defer to the expertise of cardiology, they do not gain experience and familiarity with the drug, and that causes them to feel incapable of effectively prescribing and monitoring MRAs. In contrast, cardiologists expressed comfort working with MRAs and demonstrated strong knowledge—not surprising, given their regular contact with HF patients and familiarity with published MRA clinical trials.

Similar to findings from a study of barriers to beta‐blocker and ACE inhibitor use in HF patients,^20^ noncardiology providers reported concerns about their respective “roles and responsibilities” (comanagement) and would defer to cardiology to initiate MRA therapy for patients who were comanaged. This reflected the sentiment that MRA therapy was a cardiology‐specific therapy outside of the norm of primary care. The survey findings reinforce this observation in that half of cardiology providers and 86% of PCPs felt that cardiology providers should be responsible for prescribing MRAs. This raises an important question that has not been discussed in the literature: Whose responsibility is MRA therapy? Without clarification of the roles and responsibilities of different clinical specialties in the care of patients, it will be difficult to drive adoption of MRAs. Coordination of care, especially during transitions, was seen as a barrier to MRA therapy and was described in prior studies of ACE inhibitor and beta‐blocker prescription.^20^ Although the importance of good transitions of care has received increasing attention in recent years,^21^ it remains a major barrier despite the VA's renowned electronic patient medical record system. Furthermore, we noted that because many transitions occur at hospital discharge, when patients are primarily cared for by rotating VA resident physicians, there may be opportunities for improved training regarding VA‐specific clinical care processes and resources.

Providers also cited a need for protocols to specify clinician responsibility for MRA therapy, and they desired resources to improve MRA monitoring. At present, a potential model for such a system is the anticoagulation clinic. In many VA medical centers, clinical protocols mandate automatic cancellation of warfarin prescription if patients fail to complete monthly lab tests. Without similar tools to support implementing MRA therapy, providers described uncertainty about and reluctance to prescribe without a “safety net” in place.

Other barriers such as perceived patient nonadherence, adverse effects of MRAs, and concerns about polypharmacy and competing comorbidities have been described as barriers to implementation of various guidelines, including ACE inhibitor and beta‐blocker use in HF.^20^ Despite these known patient‐related factors that may limit guideline adherence, this study supports past research that suggests current performance measurement systems do not effectively capture these barriers and tend to focus on standard medical contraindications rather than other contextual factors.^22^

The question emerges: What is needed to improve MRA prescribing and associated potassium monitoring? Health systems need to implement multimodal strategies that target all relevant barriers. They need to improve knowledge of MRAs among clinicians, including resident physicians, and support hands‐on experience with MRA use. Clinicians need adequate time and support infrastructure to address all patient care needs, not only the most pressing crisis‐driven demands. This could include system‐level tools, such as laboratory reminder systems and pharmacist‐run high‐risk medication initiation and titration clinics, to support safe use of MRAs. Medical centers need to improve handoffs between providers and to establish norms and expectations for patient care. They must establish who is responsible for initiating HF therapy, a seemingly elementary but essential activity. They need to monitor and analyze patient‐level barriers to develop local interventions that address these barriers in an individualized way.

As with all studies, methodological limitations should be noted. This was a single‐center study; therefore, generalizability remains to be confirmed. The credibility of our findings, unique to the prescription of MRAs, is strengthened by the substantial consistency with studies regarding physician barriers to ACE inhibitor and beta‐blocker use in HF patients.^20^, ^22^ We addressed our topic from a variety of complementary methods, including a survey, focus groups, and member check interviews, to arrive at our conclusions. Although a limitation of qualitative research is the use of nonrandom sampling, we used convenience and maximum variation sampling to increase the heterogeneity of perspectives captured in our analysis. The survey response rate was low, but this could be expected because respondents were asked to complete the survey only if interested in participating in a focus group. Furthermore, the single largest group of potential respondents were medical residents (n=99), who were spread across the VA and a larger community hospital. In addition, we do not have information on the survey respondents' individual MRA‐prescribing behaviors, which would be useful to correlate with their knowledge and awareness of MRAs. We know that our VA medical center has MRA‐prescribing rates (unpublished data) similar to national VA MRA prescribing rates^9^; therefore, it may reasonably represent other VA medical centers. Finally, some participants may have been minimally involved in the care of patients with HF; however, all participants had clinical roles and would be expected to have basic knowledge of HF.

This study is the first to provide a framework for understanding the barriers that limit the adoption of MRAs in HF care. We found that reasons for the MRA gap are complex and interconnected. We suggest using these identified barriers as a roadmap to implement multilevel interventions for patients, providers, and health systems to improve the quality and safety of MRA prescription.

## Sources of Funding

This study was funded by the Veterans Administration Health Services Research and Development Rapid Response Project mechanism (grant number RRP 12‐456). This work does not necessarily represent the official views of the United States government or the US Department of Veterans Affairs. Kavalieratos was supported by K12HS022989 from the Agency for Healthcare Research and Quality, and a Junior Faculty Career Development Award from the National Palliative Care Research Center.

## Disclosures

None.
